# Role of combined use of mean platelet volume-to-lymphocyte ratio and monocyte to high-density lipoprotein cholesterol ratio in predicting patients with acute myocardial infarction

**DOI:** 10.1186/s13019-023-02268-4

**Published:** 2023-05-06

**Authors:** Jianlei Cao, Rui Li, Tao He, Lin Zhang, Huixia Liu, Xiaoyan Wu

**Affiliations:** 1 Department of Cardiology, Zhongnan Hospital of Wuhan University, Wuhan, 430071 Hubei China; 2 Department of Geriatrics, Zhongnan Hospital of Wuhan University, Wuhan, 430071 Hubei China

**Keywords:** Mean platelet volume-to-lymphocyte ratio (MPVLR), Monocyte to high-density lipoprotein cholesterol ratio (MHR), Gensini score, Grace score, Acute myocardial infarction (AMI)

## Abstract

**Background:**

Atherosclerosis and thrombosis play important roles in the pathophysiology of acute coronary syndrome, with platelet activation and inflammation as the key and initiative factors. Recently mean platelet volume-to-lymphocyte ratio (MPVLR) and monocyte to high-density lipoprotein cholesterol ratio (MHR) have emerged as new prognostic indicators of cardiovascular diseases. However, the predicting effect of the combination of MPVLR and MHR in myocardial infarction has not been reported.

**Objective:**

The aim of this study was to investigate the usefulness of combination of MPVLR and MHR in predicting patients with AMI.

**Methods:**

375 patients who had chest pain or stuffiness were retrospectively enrolled in this study. According to the results of coronary angiography and cardiac troponin, patients were divided into AMI group (*n* = 284) and control group (*n* = 91). MPVLR, MHR, Gensini score and Grace score were calculated.

**Results:**

MPVLR and MHR were significantly higher in AMI group than that in control group (6.47 (4.70–9.58) *VS* 4.88 (3.82–6.44), 13.56 (8.44–19.01) *VS* 9.14 (7.00–10.86), *P* < 0.001, respectively). Meanwhile, both were positively correlated with Gensini score and Grace score. Patients with a high level of MPVLR or MHR had an increased risk for AMI (odds ratio (OR) = 1.2, 95% confidence interval (CI) 1.1–1.4, OR = 1.2, 95% CI 1.2–1.3). Combination of MPVLR and MHR identified a greater ROC area than its individual parameters (*P* < 0.001).

**Conclusion:**

Both MPVLR and MHR are independent predictors of AMI. Combination of MPVLR and MHR had higher predicting value in AMI, and thus appears to be a new risk factor and biomarker in the evaluation of risk and severity of atherosclerosis in AMI.

**Supplementary Information:**

The online version contains supplementary material available at 10.1186/s13019-023-02268-4.

## Introduction

Inflammation and platelet activation are the two major pathophysiological mechanisms in AMI [1, 2]. Atherosclerotic diseases produce chronic low grade inflammation and they are characterized with increased levels of inflammatory markers [3]. Platelet activation and hyperreactivity plays a key role in the process of intravascular thrombus [4, 5]. Mean platelet volume, which is a clinically available parameter, is a marker of platelet activation [6], and it has also been linked with inflammation in many conditions such as infections [7], hypothyroidism [8], cancer [9], vertebral disc conditions [10], obesity [11], rheumatoid arthritis [12], and type 2 diabetes [13]. On the other hand, lymphocyte count is negatively correlated with inflammation, and low lymphocyte count is associated with worse outcome in patients with coronary artery disease [14, 15]. Recently, platelet volume-to-lymphocyte ratio (MPVLR) has emerged as a new indicator of thrombus burden. Elevated MPVLR value is an independent risk factor of early and late mortality in diabetic patients with STEMI [16]. MPVLR, has also been reported to be associated with certain inflammatory disease including diabetic kidney disease [17], frailty [18], and infection [19].

On the other hand, monocytes, as one of the most important components of inflammation and immune system, also take part in the inflammatory response at the vulnerable plaque sites [20]. High-density lipoprotein cholesterol (HDL-C) exerts anti-inflammatory, antioxidant, and anti-thrombotic effects [21, 22]. Some researchers found that HDL-C could modulate monocyte activation and prevent monocytes recruitment to the artery wall [23]. Monocyte to high-density lipoprotein cholesterol ratio (MHR) has recently emerged as an indicator of inflammation and oxidative stress, and has been reported as a new predictor and prognostic indicator of coronary artery disease [24–26], and sepsis as well [27].

The aim of this study was to investigate whether the combination of MPVLR and MHR had the incremental value for predicting AMI, compared with using MPVLR or MHR alone.

## Patients and methods

### Study population

This is a retrospective study. The study protocol was approved by the ethic committees of Zhongnan hospital, Wuhan, China (Ethic No. 2021053). Informed consent was waived. From February 2021 to August 2022, patients with chest pain who admitted to Department of Cardiology, Zhongnan Hospital, Wuhan University were retrospectively screened. AMI was diagnosed based on the criteria recommended by the current American College of Cardiology guideline [28]. Patients with a history of coronary revascularization, inflammatory or hematological disease, renal or hepatic insufficiency, malignancy, myocarditis and cardiomyopathy were excluded. Clinical information and demographical data were collected from medical records.

### Biochemical and hematological parameters

The venous blood was drawn from antecubital vein after an overnight fasting and then delivered to the department of Clinical Laboratory of Zhongnan Hospital for biochemical and hematological parameters detection. MHR was calculated as monocyte count divided by the HDL cholesterol count [25], MPVLR was calculated as MPV divided by lymphocyte count (10^3^/mm^3^) [16].

### Gensini score and Grace score assessment

Evaluation of coronary angiograms and determination of Gensini score were determined separately by three cardiologists who were blinded to the laboratory and clinical data of patients. Grace score was calculated by another cardiovascular physician. Both of the indexes were based on the classic Gensini score system and GRACE risk score system [29, 30].

### Statistical analysis

Quantitative data are presented as mean ± standard deviation (SD) or medians with interquartile ranges (lower and upper quartiles) according to their normality. The Kolmogorov–Smirnov normality test was used to examine whether variables are normally distributed. Qualitative data are presented as frequencies (*n*%). Student t-test was used to compare continuous variables conforming to normal distribution between groups, otherwise Mann–Whitney test was used. Chi-Square test was used to compare proportions. Pearson or Spearman coefficients were used for evaluating the correlations. Multivariate logistic regression analyses were used to identify the predictive variables for presence of AMI. The area under the receiver operating characteristics curve (AUC) were used to quantify the overall diagnostic value of MPVLR, MHR and combination of the two ratios. Data were analysed using SPSS 17.0 (SPSS Inc., Chicago, IL, USA) software. Statistical significance was defined as *P* < 0.05.

## Results

A total of 375 patients with chest pain were consecutively included in this study. Then patients were divided into two groups, AMI group and no-AMI group. The baseline characteristics, including Clinical, demographical, biochemical, and hematologic measurements of the study population are presented in Tables 1 and 2, respectively. There are no differences in the two groups for age, Body Mass Index (BMI), systolic blood pressure (SBP), diastolic blood pressure (DBP), heart rate (HR), hypertension, hypercholesterolemia, Diabetes mellitus and erythrocytes count, hemoglobin value, platelet count, MPV, creatinine, Triglyceride (TG) and Low-Density Lipoprotein Cholesterol (LDL-C) (*P* > 0.05, respectively). Patients in AMI group had higher percentage of male and history of smoking and drinking (*P* < 0.001, respectively). Patients in AMI group also spend longer time in hospital. Gensini score and Grace score were higher in AMI group than that in normal group (*P* < 0.001, respectively), the days of hospital stay was longer in AMI group than normal group (*P* < 0.001). Patients in AMI group had significantly greater levels of leukocytes, monocytes, MPVLR, MHR, TC values (*P* < 0.01) and UA (*P* < 0.05). In contrast, AMI group had lower lymphocytes (*P* < 0.05), HCT (*P* < 0.01) and HDL-C (*P* < 0.001).

**Table 1:** Patients' baseline and clinical characteristics

	AMI group(*n* = 284)	Normal group(*n* = 91)	*P* value
Age (years)	61.27 ± 12.01	59.10 ± 11.96	0.08
Male [n(%)]	237(83.45%)	51(56.04%)	0.00***
BMI (kg/m^2^)	24.06 ± 1.87	24.54 ± 2.01	0.12
SBP (mmHg)	129.48 ± 25.40	130.24 ± 21.25	0.22
DBP (mmHg)	76.99 ± 16.20	78.01 ± 14.26	0.09
HR (beats/minute)[median(IQR)]	75.00(67.00–84.00)	73.00(70.00–79.00)	0.25
Smoking [n(%)]	177(62.32%)	23(25.27%)	0.00***
Drinking [n(%)]	109(38.38%)	18(19.78%)	0.00**
Hypertension [n(%)]	157(55.28%)	51(56.04%)	0.90
Hypercholesterolemia [n(%)]	155(54.58%)	46(50.55%)	0.50
Diabetes mellitus [n(%)]	56(19.72%)	15(16.48%)	0.49
Gensini score [median(IQR)]	53.00(28.60–82.75)	2.00(0.00–6.00)	0.00***
Grace score [median(IQR)]	138.00(118.00–162.00)	93.00(76.00–107.00)	0.00***
Hospitalstay(days) [median(IQR)]	11.00(8.00–14.00)	7.00(5.00–9.00)	0.00***

*BMI* body mass index; *SBP* systolic blood pressure; *DBP* diastolic blood pressure; *HR* heart rate; *IQR* interquartile range

Compared with normal group, **P < 0.01, ***P < 0.001

**Table 2:** Laboratory findings

	AMI group(*n* = 284)	Normal group(*n* = 91)	*P* value
Leukocytes (10^3^/mm^3^) [median(IQR)]	10.29(8.00–12.48)	6.20(5.20–7.21)	0.00****
Erythrocytes (10^6^/mm^3^)	4.46 ± 0.60	4.40 ± 0.48	0.39
Hemoglobin (g/L) [median(IQR)]	137.00(127.00–146.70)	137.00(131.30–144.90)	0.46
Platelet count (10^3^/mm^3^)	198.85 ± 56.67	192.30 ± 53.43	0.33
Lymphocytes (10^3^/mm^3^)	1.55 ± 0.036	1.88 ± 0.44	0.02*
Monocytes (10^9^/ul)	573.54 ± 280.38	443.56 ± 45.11	0.00***
HCT [median(IQR)]	40.71(38.02–43.78)	41.80(40.60–45.20)	0.00**
NLR [median(IQR)]	5.4(3.39–9.2)	1.93(1.36–2.71)	0.00***
MPV(fl) [median(IQR)]	9.15(8.23–10.45)	8.80(8.00–10.10)	0.20
MPVLR [median(IQR)]	6.47(4.70–9.58)	4.88(3.82–6.44)	0.00***
MHR [median(IQR)]	13.56(8.44–19.01)	9.14(7.00–10.86)	0.00***
Creatinine (umol/L) [median(IQR)]	73.60(60.33–87.98)	72.60(61.70–84.20)	0.96
UA (umol/L)	360.30 ± 96.82	331.58 ± 101.69	0.02*
TC (mmol/L)	4.85 ± 0.84	4.47 ± 1.00	0.00**
TG (mmol/L) [median(IQR)]	1.54(1.07–2.38)	1.58(1.15–2.10)	0.81
LDL-C (mmol/L)	2.85 ± 0.86	2.83 ± 0.74	0.80
HDL-C (mmol/L) [median(IQR)]	1.00(0.88–1.18)	1.22(1.04–1.40)	0.00***
hsCRP	11.89 ± 25.8	1.51 ± 1.67	0.00***

*HCT* hematocrit; *MPV* mean platelet volume; *MPVLR* mean platelet volume-to-lymphocyte ratio; *NLR* neutrophil–lymphocyte ratio; *UA* uric acid; *TC* total cholesterol; *TG* triglyceride; *LDL-C* low-density lipoprotein cholesterol; *HDL-C* high-density lipoprotein cholesterol; *MHR* Monocyte to HDL ratio; *hsCRP* High-sensitivity C-reactive protein

Compared with normal group,*p < 0.05;**p < 0.01;***p < 0.001

The correlation analysis was shown in Additional file 1: Fig. S1, MPVLR and MHR were significantly associated with Gensini score (r = 0.201 and 0.143, respectively) and Grace score (r = 0.268 and 0.116, respectively); After adjusting for gender, age, BMI, smoking, hypertension and diabetes, multivariate logistic regression analysis was shown in Table 3, MPVLR and MHR were significantly associated with AMI (OR = 1.41, 95% CI 1.24–1.60 *VS* OR = 1.24, 95% CI 1.15–1.33).

**Table 3:** Multivariate logistic regression analysis for association of MPVLR, MHR with AMI

	Odds ratio	Standard error	P value	OR 95% CI	
				Lower	Upper
MPVLR	1.41	0.09	0.00	1.24	1.60
MHR	1.24	0.05	0.00	1.15	1.34
Male	1.49	0.58	0.31	0.70	3.18
Age	1.06	0.02	0.00	1.02	1.09
BMI	0.86	0.07	0.08	0.74	1.01
Smoke	7.84	3.19	0.00	3.53	17.41
HBP	1.12	0.37	0.74	0.58	2.13
Diabetes	1.50	0.61	0.31	0.68	3.32
Hyperlipide~a	1.50	0.49	0.21	0.80	2.84
Cons	0.01	0.02	0.04	0.00	0.79

*MPVLR* mean platelet volume-to-lymphocyte ratio; *MHR* Monocyte to HDL ratio; *HDL-C* high- density lipoprotein cholesterol; *CI* confidence interval; *OR* odds ratio

For further investigation, we divided all cases into subgroups according to quartile value of MPVLR and MHR as follows: Group A: MPVLR < 4.43; Group B: 4.43 ≤ MPVLR < 5.99; Group C: 5.99 ≤ MPVLR < 8.96; Group D: MPVLR ≥ 8.96; Group E: MHR < 7.96; Group F: 7.96 ≤ MHR < 11.29; Group G: 11.29 ≤ MHR < 17.44; Group H: MHR ≥ 17.44. Patients in top quartile of MPVLR had an OR of 4.48 (95% CI 2.10–9.53, Table 4) compared with the value bottom quartile OR of 1.33 (95% CI 0.73–2.44), while MHR in top quartile had an OR of 12.58 (95% CI 4.24–37.37, Table 5) compared with bottom quartile OR of 0.85 (95% CI 0.47–1.54, Table 5).

**Table 4:** Logistic regression analysis for association of the quartile groups of MPVLR with AMI

	Odds ratio	Standard error	P value	OR 95% CI	
				Lower	Upper
B	1.33	0.41	0.36	0.73	2.44
C	2.85	0.99	0.00	1.44	5.65
D	4.48	1.72	0.00	2.10	9.53
Cons	1.69	0.36	0.01	1.11	2.56

*MPVLR* mean platelet volume-to-lymphocyte ratio; *MHR* Monocyte to HDL ratio; *HDL-C* high- density lipoprotein cholesterol; *CI* confidence interval; *OR* odds ratio

**Table 5:** Logistic regression analysis for association of the quartile groups of MHR with AMI

	Odds ratio	Standard error	P value	OR 95% CI	
				Lower	Upper
F	0.85	0.26	0.600	0.47	1.54
G	2.69	0.94	0.005	1.35	5.35
H	12.58	6.99	0.000	4.24	37.37
Cons	1.79	0.39	0.008	1.17	2.74

**Fig. 1:**
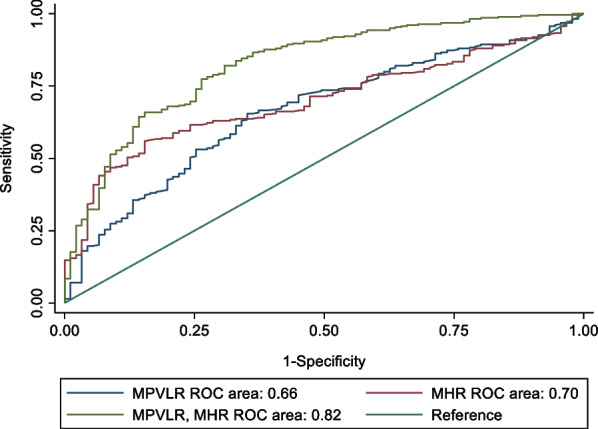
Receiver-operating characteristic analysis of MPVLR, MHR and combination of MPVLR and MHR for AMI

Finally, we used the area under the curve (AUC) of ROC curve to determine the predictive values of MPVLR and MHR. Combination of MPVLR and MHR identified a greater ROC area than its individual parameters (*P* < 0.001, Table 6 and Fig. 1, respectively).

**Table 6:** Area under the receiver operating characteristics curve for AMI

	Area under the ROC curve	95% CI	
		Lower	Upper
MPVLR	0.66	0.60	0.72
MHR	0.70	0.64	0.75
Combination of MPVLR and MHR	0.82	0.77	0.87

*MPVLR* mean platelet volume-to-lymphocyte ratio; *MHR* monocyte to HDL ratio; *CI* confidence interval

## Discussion

Prognostic values of MPV, platelet to lymphocyte ratio (PLR), MPVLR and MHR have been investigated in AMI. However, the predicting effect of the combination of the two in myocardial infarction has not been reported. Therefore, we evaluated the combination of MPVLR and MHR in predicting AMI.

Inflammatory and thrombotic milieu play an important role in acute coronary syndrome (ACS) [1, 2]. Inflammation takes part in all stages of atherosclerosis, from initiation to progression, eventually leading to thrombus formation [31]. Lymphocyte is one of the key part of chronic inflammation during atherosclerosis. They infiltrate the ischemic myocardium and secrete interleukin-10, which recruits mononuclear cells. Low lymphocyte count in patients with ACS is associated with worse prognosis in AMI [32]. On the other hand, platelets play a significant role in thromboembolic events and in the atherosclerotic process. Platelets interact with endothelial cells, secreting chemokines which induce the migration and activation of monocytes and neutrophils. Activated platelets can further induce platelet adhesion and aggregation, and thus initiate plaque formation [33]. Mean platelet volume (MPV), representing platelet size, correlating with its activity, is available in clinical settings [5]. Previous studies showed that high value of MPV leads to increased impaired reperfusion and long term mortality in AMI, and thus these patients should be given more effective antiplatelet therapy [34, 35]. MPVLR, based on the interaction between platelets activity and inflammatory system, indicated a greater thrombus burden, has been noted as an independent risk factor of early and late mortality [16]. Diabetes mellitus is the most common risk factor of heart diseases. Studies showed that MPV was significantly elevated in type 2 diabetic patients compared to control subjects [13]. Both MPV and MPVLR have been suggested to be a surrogate marker of frailty in type 2 diabetes mellitus [18, 36]. In addition, MPV and MPVLR have also been associated with chronic processes such as hepatosteatosis [37], hypothyroidism [8], suggesting an important role of these markers in chronic inflammatory disease. Our results showed the value of MPVLR in AMI group were higher than that in normal group, which eco the previous findings. Logistic regression showed that when levels of MPVLR increased, the Odds Ratio of AMI increased significantly. In ICU patients, MPV levels could be an indicator for worse outcome [38]. Whether these markers could be used as prognostic markers in AMI worth further study.

Meanwhile, circulating monocytes are key cell type for atherosclerotic plaque formation. They interact with platelets and endothelial cells, leading to aggravation of inflammatory, pro-thrombotic pathways, in atherosclerotic plaque formation process. Monocytes differentiate into foam cells by taking up oxidized LDL, so monocyte count was found to be a predictor for new plaque development [39]. HDL-C molecules suppress monocyte activities, interrupt differentiation of monocytes to macrophages, counteract migration of macrophages, remove cholesterol from these cells and moreover inhibit LDL-C oxidation [40–42]. MHR has been considered as a marker of inflammatory status and oxidative stress as well as risk factor for coronary artery disease. Recent study reported that MHR was associated with increased risk of MACE and mortality in ACS patients, and could serve as a potential prognostic indicator for ACS [43]. In STEMI patients who underwent primary PCI, MHR is positively associated with a higher in-hospital mortality [44]. Our results showed similar results that the value of MHR in AMI group were higher than that in normal group, and that levels of MHR is an independent risk factor of AMI by logistic regression.

Gensini score represents the severity of coronary stenosis, which can not only evaluate coronary artery lesions and but also the risk of cardiovascular and cerebrovascular events in patients with CAD [45, 46]. Grace score has been used to predict in-hospital mortality of patients with ACS. In this study, we found the close relationship between MPVLR, MHR and the above two scores. Other study also found a close relationship between MHR and Gensini score, and also SYNTAX score. MHR is also related with higher risk for inhospital major adverse cardiac events [24, 25]. These data suggest that MPVLR and MHR could be the indicators of the severity of coronary lesion and patients’ prognosis.

Finally, we evaluated the predictive value of MPVLR and MHR with AMI and found that the combination of the two parameters was more efficient in predicting AMI than the individual ones. This suggests that the combination of MPVLR and MHR, which connects inflammation and platelet activity in the pathological process of thrombosis in patients with AMI, can supply more evidence of risk for the severity of atherosclerosis stenosis and might be used as a new biomarker in predicting AMI.

There are a few limitations in our study that should be considered. This was a single center and retrospective study. We did not take into account the other markers such as IL-6, CRP, thrombomodulin and APoA1 which are also involved in the process. In addition, we haven’t take into account different pathological types of AMI. The relationship between MPVLR and MHR and different types of AMI might be discussed in future studies.

In conclusion, as combined values of MPVLR and MHR better predicted risk and severity of atherosclerosis in AMI than their individual values, it appeared to be a new predictor and can be commonly used as a biomarker in evaluation of risk and severity of atherosclerosis in AMI.

## Supplementary Information

**Additional file 1**. Correlation between MPVLR, HHR and Gensini score, Grace Score.

## Data Availability

The dataset supporting the conclusions of this article is available from Xiaoyan Wu (e-mail:wuxiaoyan299@whu.edu.cn) upon reasonable request and with permission of Wuhan University Zhongnan Hospital.

## Abbreviations

MPVLR: Mean platelet volume-to-lymphocyte ratio
MHR: Monocyte to high-density lipoprotein cholesterol ratio
MPV: Mean platelet volume
PLR: Platelet to lymphocyte ratio
AMI: Acute myocardial infarction
AUC: Area under the curve
BMI: Body Mass Index
SBP: Systolic blood pressure
DBP: Diastolic blood pressure
HR: Heart rate
PLR: Platelet to lymphocyte ratio
HDL-C: High-density lipoprotein cholesterol
Low-C: Low-density lipoprotein cholesterol
