# Supplementary figures and images for: Role of combined use of mean platelet volume-to-lymphocyte ratio and monocyte to high-density lipoprotein cholesterol ratio in predicting patients with acute myocardial infarction
Source: J Cardiothorac Surg. 2023 May 6;18:172. doi: 10.1186/s13019-023-02268-4 (PMC10163726; doi:10.1186/s13019-023-02268-4)

Figure s1. Correlation between MPVLR, HHR and Gensini score, Grace Score.

(A)

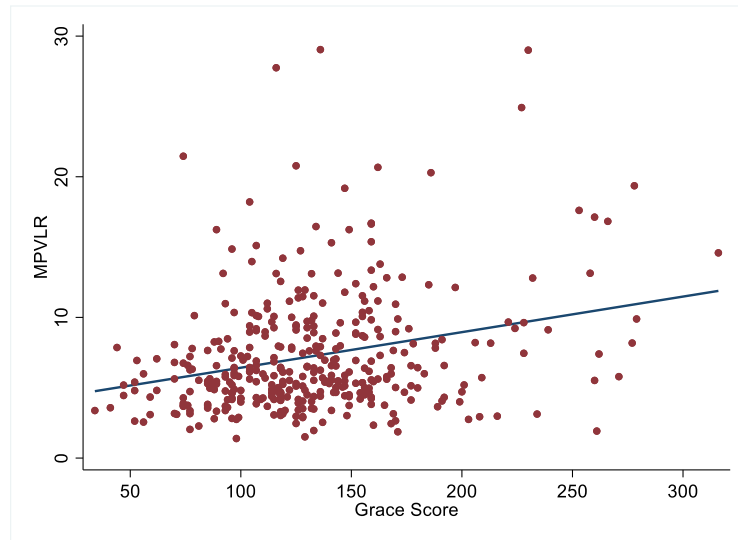

(B)

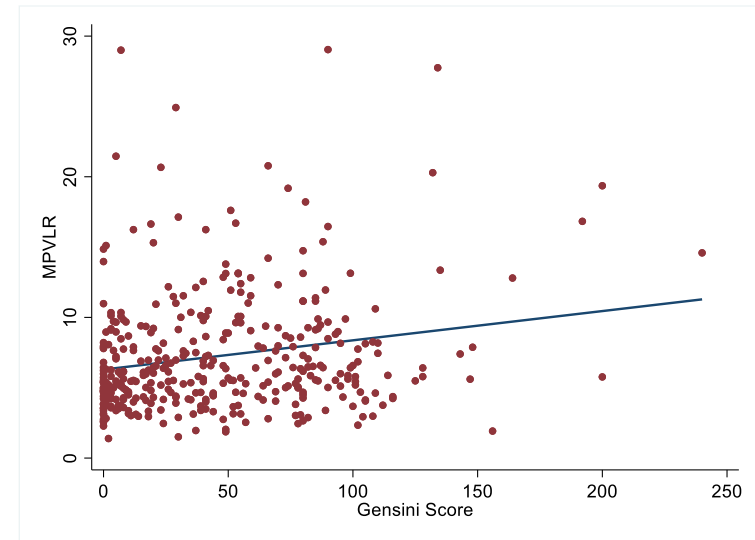

(C)

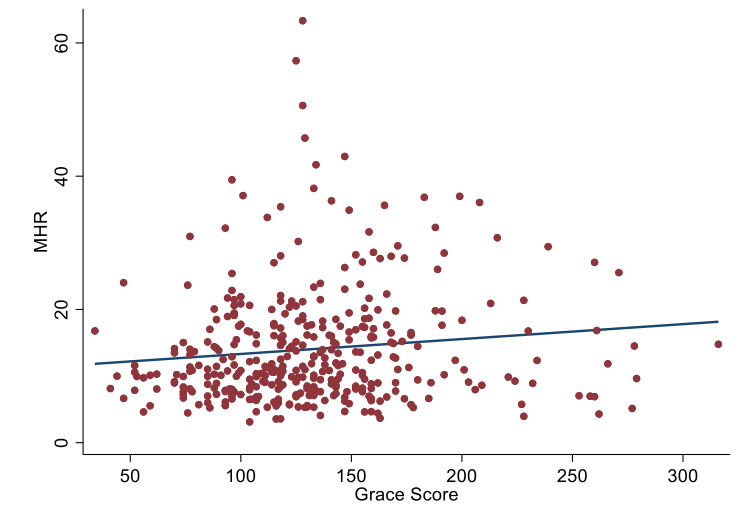

(D)

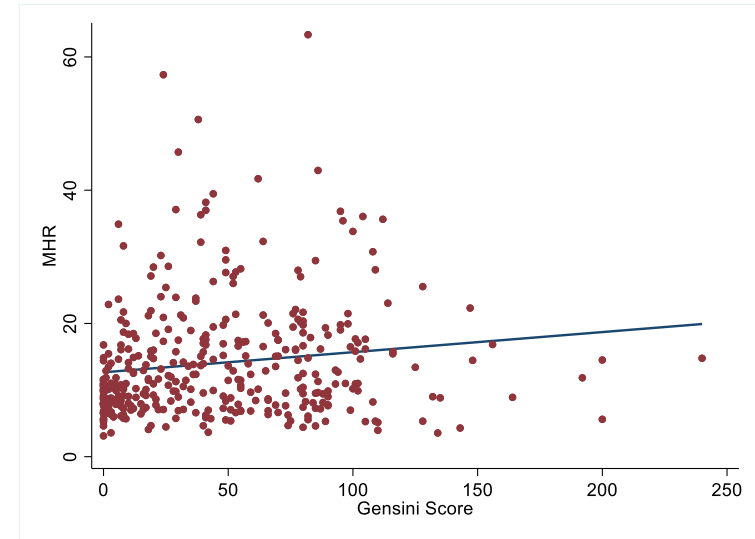

Supplement: Supplementary file 1 — Additional file 1. Correlation between MPVLR, HHR and Gensini score, Grace Score. [file 13019_2023_2268_MOESM1_ESM.pdf]
